# Intracranial complications after surgical removal of nasal polyps

**DOI:** 10.1093/jscr/rjab109

**Published:** 2021-04-27

**Authors:** Agnieszka Nowacka, Maciej Śniegocki, Wojciech Smuczyński, Kamila Woźniak-Dąbrowska

**Affiliations:** 1 Department of Neurosurgery, Nicolaus Copernicus University Collegium Medicum in Bydgoszcz, Bydgoszcz, Poland; 2 Department of Physiotherapy, Nicolaus Copernicus University Collegium Medicum in Bydgoszcz, Bydgoszcz, Poland

## Abstract

Acute otitis media can be caused by viruses (nonsuppurative inflammation) and bacteria (suppurative inflammation). The suppurative inflammation is a risk factor for endocranial complications, including subdural empyema burdened with a high mortality. The authors present a clinical case of a 67-year-old man treated by surgery for a subdural empyema in the course of the acute suppurative otitis media.

## INTRODUCTION

Sinusitis complications occur in both acute and chronic rhinosinusitis [1]. A direct risk to the patient's life is intracranial complications, including meningitis and inflammation of the brain tissue with its focal infection, such as a brain abscess or less frequently supra- and subdural empyema [2, 3]. The vicinity of the inflamed paranasal sinuses with the anterior and posterior cranial fossa poses a potential risk of infection passing towards the skull [4]. Intracranial complications develop more often as a result of chronic sinusitis, but also by bone defects or inaccurately performed procedures from an external approach [5–7].

## CASE REPORT

**Figure 1:**
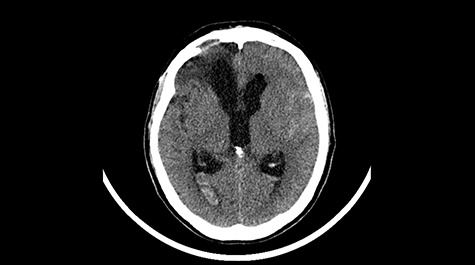
Head CT after polypectomy—presence of blood in fluid spaces, widening of the ventricular system, presence of air in the frontal horns of the lateral ventricles, features of cerebral edema. (Department of Neurosurgery Neurotraumatology and Pediatric Surgery own material).

**Figure 2:**
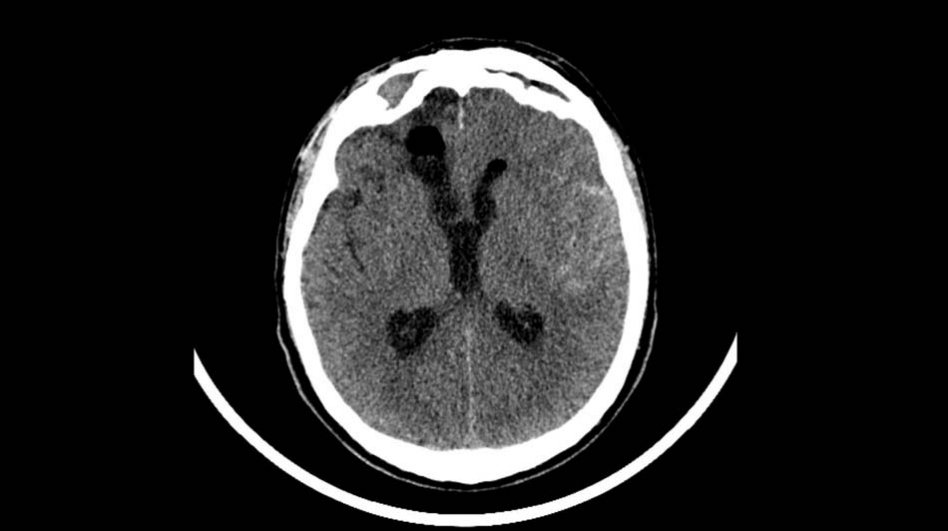
Head CT—state before VP shunt implantation. (Department of Neurosurgery own material).

A 49-year-old patient presented after surgical treatment of nasal polyps, with headache and consciousness disorders (behavioral changes, drowsiness and allophenic orientation disorders). Computed tomography (CT) scan showed (Fig. 1) a presence of blood in fluid spaces, widening of the ventricular system (without active hydrocephalus features) and presence of air in the frontal horns of the lateral ventricles and features of cerebral edema. Due to the subarachnoid hemorrhage, an angio-CT and CT scan was performed, showing no vascular malformation. The patient's condition deteriorated with a drop in Glasgow Coma Scale (GCS) to 9, strongly expressed meningeal syndrome and a fever of > 38°C. In the performed cerebrospinal fluid examination a typical picture for bacterial infection, cultures negative. Treatment was implemented in accordance with the neuro-infections algorithm. Clinical and laboratory features of neuroinfections have withdrawn. The neurological condition of the patient improved to GCS 11. Control head CT (Fig. 2) showed enlargement of the ventricular system with cerebrospinal fluid transudation.

**Figure 3:**
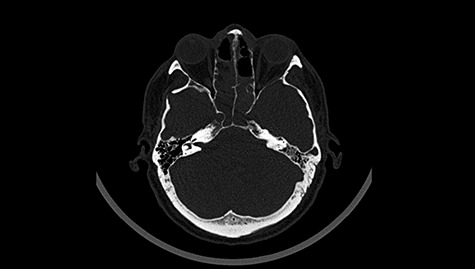
Head CT after polypectomy—seen defects of the upper left orbital wall. (Department of Neurosurgery own material).

**Figure 4:**
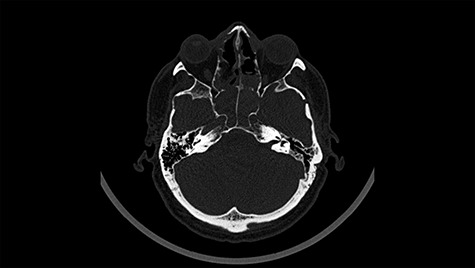
Head CT after polypectomy—seen sphenoid sinus and both maxillary sinuses filled with blood. (Department of Neurosurgery own material).

**Figure 6:**
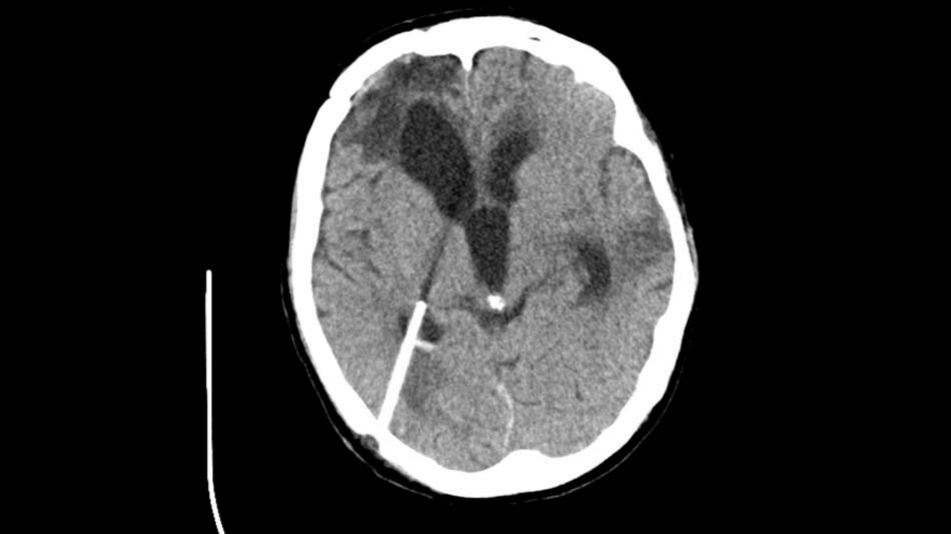
Head CT—state after VP shunt implantation. (Department of Neurosurgery own material).

**Figure 7:**
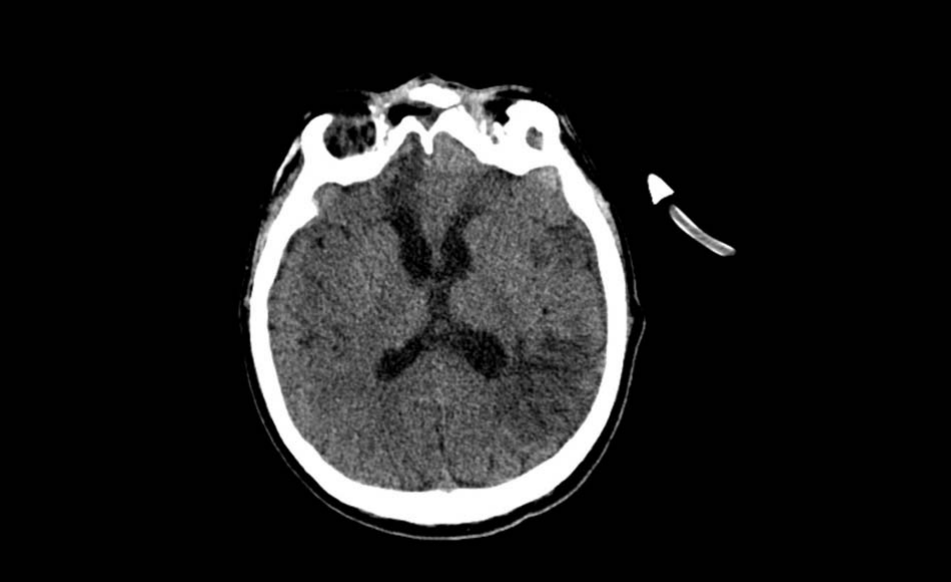
Head CT—seen absorption of air above the right orbital, the image without features of fresh bleeding, a slightly narrower ventricle. (Department of Neurosurgery own material).

The patient was qualified for VP shunt implantation, during which elevated cerebrospinal fluid pressure, above 300-mm H_2_O, was found. Further small improvement in neurological status was obtained. Periodically we observed increased body temperature and increase in laboratory exponents of inflammation (after an earlier decrease to values close to normal). There were several convulsive epileptic seizures with loss of consciousness with no response to benzodiazepines, therefore thiopental was administrated, patient was intubated and ventilation therapy started. In the course of further hospitalization the body temperature increased to over 39°C, with an increase in inflammatory markers. Positive blood cultures (*Staphylococcus epidermidis*), urine (*Klebsiella pneumoniae* and *Pseudomonas aeruginosa*) and bronchoalveolar lavage (*P. aeruginosa*) were obtained. Empirical treatment was applied followed by targeted antibiotic therapy. There was a gradual improvement in the general and neurological status—up to GCS 14. Negative blood, urine and mini-bronchoalveolar lavage (mini-BAL) cultures were obtained. The tracheostomy tube was removed and the tracheostomy hole was closed. A few days later there were two epileptic seizures and further deterioration in the general and neurological condition, therefore the patient was re-intubated, mini-BAL was performed. The next day in the morning hours, seizures occurred several times, initially regressing after administration of Relanium, then not responding to this drug. Depakine administrated in the i.v pump. A day later, in the morning, there was a seizure again. Thiopental was administrated, ventilotherapy started. A lumbar puncture was performed showing a cerebrospinal fluid without inflammatory features. Finally, the patient condition improved slowly overtime.

## DISCUSSION

The patient has been suffering from chronic sinusitis for years. Three years earlier, he was treated surgically because of a brain empyema in the right frontal lobe, resulted from sinusitis. He had a maxillary, right frontal and sphenoid sinus operated, which significantly influenced the subsequent course of the disease. An important role in the development of intracranial complications in the course of chronic rhinosinusitis is the inflammation of the spongious substance layer of the flat skeletal bones of the skull. The slow flow of blood in the diploe and close contact of Bracheta veins with the mucous membrane of the inflamed sinus walls, makes their colonization easier for bacterial pathogens, which leads to the formation of infected thrombi in their lumen [8]. Osteomielitic foci of flat bone of the skull arise, which leads to the formation of extensive defects of bone tissue [2]. Thrombophlebitis of the diploe can lead to bone destruction and intracranial complications (e.g. subdural empyema), which requires an urgent surgical treatment [4].

In described case, a head CT scan after polypectomy confirmed the subarachnoid hemorrhage (Fig. 1). In addition, the presence of air in the ventricular system was visible, and presence of defects of the upper left orbital wall and ethmoid on both sides (Fig. 3). In the CT scan sphenoid sinus, frontal and ethmoidal sinuses on both sides and right maxillary sinus airless, filled with blood and soft tissue. The changes also concerned the lateral part of the left maxillary sinus (Fig. 4).

The present bone defects were most probably caused by the development of osteomalytic foci as a consequence of the spread of the inflammatory process within the diploe of flat skeletal skull bones, in the course of chronic rhinosinusitis. Both underestimating the severity of complaints in the course of chronic rhinosinusitis and delaying the implementation of surgical treatment led to massive bone destruction, which had an impact on the development of further complications after polypectomy (including subarachnoid hemorrhage). Performed angio-CT excluded any malformations and the probable cause of subarachnoid hemorrhage due to rupture of the aneurysm. Considering the radiological picture and the condition of the patient, it can be concluded that the persistent inflammatory process within sinuses led to dehsomination and subsequent bleeding into the subarachnoid space through discontinuity in the bone structure of the sinuses and orbits.

Current features of neuroinfection indicated that the bacterial etiological agent penetrated into the inside of the cranial cavity through the bloodstream. In case of observed focal neurological symptoms, it is recommended to extend the diagnosis with an MRI scan in order to search for other changes within the central nervous system [4, 6, 7].

After improvement of patient’s neurological status, a CT scan of the head revealed enlargement of the ventricular system with the features of cerebrospinal fluid transudation (Fig. 2), thus the patient has been qualified for a VP shunt implantation (Fig. 6).

In described patient, applied pharmacological and surgical treatment brought about an improvement, which was confirmed by the CT results (Fig. 7).

## CONFLICT OF INTEREST STATEMENT

Authors declare no conflicts of interest.

## ETHICAL STATEMENT

No ethical approval is required.

## GUARANTORS

All authors are guarantors.
